# P02-019 - Detection of risk factors for AA-amyloidosis

**DOI:** 10.1186/1546-0096-11-S1-A126

**Published:** 2013-11-08

**Authors:** S Németh, L Obici, S Grandemange, H Lachmann, C Oberkanins

**Affiliations:** 1 ViennaLab Labordiagnostika GmbH, Vienna, Austria; 2 Biotechnology Research Laboratories, IRCCS Fondazione Policlinico San Matteo, Pavia, Italy; 3 Unité médicale des maladies auto-inflammatoires, Hôpital Arnaud de Villeneuve, Montpellier, France; 4 National Amyloidosis Centre, UCL Medical School, London, UK

## Introduction

Systemic reactive (AA) amyloidosis represents the most important complication within TNF receptor associated periodic syndrome (TRAPS), familial Mediterranean fever (FMF) and other autoinflammatory syndromes, progressively leading to endstage renal failure. The homozygous condition of the serum amyloid A (SAA) variant *SAA1.1* is significantly associated with the occurrence of AA amyloidosis in TRAPS patients. Likewise in FMF patients the *MEFV* mutation c.2080A>G (M694V) correlates with amyloidosis and the *SAA1.1/SAA1.1* genotype increases clinical severity (age at disease onset, amyloidosis, arthritis).

## Objectives

To develop a reverse-hybridization assay (Amyloidosis StripAssay) for detection of (1) genetic markers modulating the risk of developing AA amyloidosis in TRAPS and FMF [c.209C>T, c.224T>C (*SAA1*); c.2080A>G (*MEFV*)], as well as (2) mannose-binding lectin 2 (*MBL2*) and *TNFRSF1A* variants, which are suspected to be modulating factors in TRAPS [g.4447 C>G, g.4776 C>G, g.5000 C>T, c.154C>T, c.161G>A, c.170G>A (*MBL2*); c.473-33C>T (*TNFRSF1A*)].

## Methods

The Amyloidosis StripAssay is based on a multiplex PCR and hybridization of biotinylated amplicons under exactly defined stringency to a teststrip presenting a parallel array of allele-specific oligonucleotide probes. Specifically bound PCR products are detected using enzymatic colour reaction.

## Results

The specificity of the StripAssay was validated by hybridizing teststrips against PCR products obtained from plasmid clones, as well as reference DNA samples. All genetic variants covered by the StripAssay could be unambiguously identified.

## Conclusion

The Amyloidosis StripAssay proved to be a fast and reliable method for detection of genetic factors modulating the risk of developing AA amyloidosis in FMF and TRAPS. An association between *MBL2* alleles and amyloidosis is not conclusively established, but *MBL2* is of considerable diagnostic interest in closely related fields, including rheumatoid arthritis or innate immunity. Also in case of the *TNFRSF1A* intron 4 polymorphism c.473-33C>T the StripAssay could be a useful tool: the c.473-33C>T variant is involved in *TNFRSF1A* expression and an implication on the TRAPS phenotype still needs to be investigated.

## Disclosure of interest

None declared.

